# The effect of photobiomodulation therapy on fatigue and behavioural status in patients with Hashimoto’s thyroiditis

**DOI:** 10.1007/s10103-026-04864-x

**Published:** 2026-03-30

**Authors:** Sümeyye Tunç, Şükriye Leyla Altuntaş, Murat Atmaca

**Affiliations:** 1 Physiotherapy Programme, Department of Therapy and Rehabilitation, IMU Vocational School, Istanbul Medipol University, Istanbul, 34083 Turkey; 2 Department of Obstetrics and Gynecology, Faculty of Medicine, Istanbul Medipol University, Istanbul, Turkey; 3 Department of Endocrinology and Metabolism, Faculty of Medicine, Istanbul Medipol University, Istanbul, Turkey

**Keywords:** Anxiety, Depression, Fatigue, Hashimoto Thyroiditis, Photobiomodulation Therapy

## Abstract

**Purpose:**

The standard lifelong treatment for Hashimoto’s thyroiditis (HT), a chronic autoimmune disease, is levothyroxine (LT4) therapy. Despite LT4 replacement therapy, patients continue to experience persistent fatigue, deterioration in psychological and general well-being. Our study was conducted to investigate the effects of photobiomodulation therapy (PMBT) combined with LT4 replacement therapy on fatigue and behavioural status in patients with HT.

**Methods:**

Sixty patients with HT were randomised to receive active or sham PMBT twice weekly for three weeks. Clinical evaluations of the participants were performed before and at the third months after treatment. Fatigue was assessed using the Fatigue Impact and Severity Scale, sleep quality and sleepiness using the Pittsburgh Sleep Quality Index and the Epworth Sleepiness Scale, and behavioural status using the Beck Anxiety and Depression Inventory.

**Results:**

At the end of the treatment fatigue severity, sleep quality, daytime sleepiness and behavioural status were improved in both groups. In the between-group comparisons, all clinical symptom states showed a significant improvement in favour of the active group (*p* < 0.05).

**Conclusions:**

As a result, we concluded that PMBT is an effective method to reduce clinical symptoms in patients with HT.

## Introduction

Hashimoto’s thyroiditis (HT) is an autoimmune disease characterised by chronic progressive thyroid tissue damage resulting from infiltration of the thyroid gland by lymphocytes under the complex influence of genetic, environmental and endogenous factors. This autoimmune process results in gradual impairment of thyroid hormone synthesis and often leads to hypothyroidism. HT is the most common cause of primary hypothyroidism in iodine-sufficient regions and predominantly affects women, with an estimated prevalence ranging from approximately 5% to 10% in the general population [1, 2]. The underlying pathophysiology involves immune-mediated damage to thyroid follicular cells, accompanied by elevated levels of thyroid peroxidase (TPO) and thyroglobulin (Tg) antibodies, and sustained inflammatory activity. Increasing evidence indicates that oxidative stress, mitochondrial dysfunction, and dysregulated cytokine activity may further exacerbate tissue damage and contribute to persistent clinical symptoms [3].

Levothyroxine (LT4) replacement therapy remains the standard of treatment for hypothyroidism resulting from HT, normalizing thyroid function and achieving biochemical euthyroidism. However, studies have reported that in patients with overt or subclinical hypothyroidism, despite normalization of thyroid function and biochemical euthyroidism with LT4 therapy, clinical symptoms often persist and quality of life remains suboptimal. Commonly reported complaints include chronic fatigue, muscle weakness, impaired concentration, excessive sleepiness, poor sleep quality, irritability, and mood disturbances such as frequent mood swings [4, 5]. These findings indicate that hormone replacement alone may not fully address the autoimmune, inflammatory, and oxidative processes underlying HT, highlighting certain limitations of conventional medical management. Given these limitations, there is increasing interest in complementary and supportive therapeutic strategies aimed at improving patient outcomes. In addition to conventional LT4 therapy, complementary interventions such as lifestyle modifications, nutritional support, and emerging non-invasive therapies have been explored to improve clinical outcomes in patients with HT [6–8]. Among these, photobiomodulation therapy (PMBT), a non-invasive, low-risk procedure, has emerged as a promising supportive approach [9, 10].

At the cellular level, PMBT induces photochemical reactions that enhance mitochondrial respiration, increase adenosine triphosphate (ATP) production. It also modulates reactive oxygen species and activates transcription factors involved in cell survival, tissue repair, and regeneration [11]. These mechanisms reduce oxidative stress and inflammation, which are key contributors to thyroid tissue damage in HT [10]. Additionally, PMBT has been shown to influence immune function by modulating cytokine release and reducing autoantibody production, suggesting a potential mechanism for mitigating autoimmune-mediated thyroid injury [12]. Evidence from preliminary clinical studies indicates that PMBT may improve thyroid function, decrease autoimmunity markers, and enhance thyroid vascularization in HT patients [13–15]. Despite these promising findings, evidence regarding its effects on neuromuscular symptoms, fatigue, sleep quality, and behavioral outcomes remains limited. Based on the molecular mechanisms of PMBT and available preliminary clinical evidence, this study was designed to evaluate the clinical symptoms efficacy of PMBT when combined with LT4 therapy. The primary objective is to assess its effects on fatigue, sleep quality, and behavioral outcomes in patients with HT. The study also aims to provide novel data on the potential benefits of PMBT, addressing the limitations of conventional hormone replacement in managing persistent symptoms. It is hypothesized that patients receiving LT4 therapy combined with active PMBT will show greater improvements in fatigue, sleep quality, and behavioral status compared to patients receiving LT4 therapy combined with sham PMBT.

## Materials and methods

The study was conducted between January 2021 and February 2023 with patients who were followed up with a diagnosis of “Hashimoto’s Thyroiditis” by a specialist doctor in the Endocrinology and Metabolic Diseases Clinic of Kosuyolu Istanbul Medipol Hospital.

### Ethical approval

This study was approved by the Istanbul Medipol University Non-Interventional Clinical Research Ethics Committee on 27 January 2021 (Decision No: 107) and was conducted in accordance with the Declaration of Helsinki. Signed informed consent was obtained from all participants. The trial was registered at ClinicalTrials.gov (Identifier: NCT06735040).

### Participant eligibility criteria

The study included patients over the age of eighteen years who were diagnosed with HT by a specialist physician and followed up with LT4 treatment. Exclusion criteria were acute infection, use of immunosuppressants, immunostimulants and drugs that interfere with the production, transport and metabolism of thyroid hormones (e.g. corticosteroids, lithium and amiodarone), thyroid nodules, tracheal stenosis, history of serious illness (e.g. cancer, ischaemic coronary artery disease, stroke, renal and liver failure), history of exposure to ionising radiation or neoplasia in the cervical region, malignancy and thyroid surgery; cancer, ischaemic coronary artery disease, stroke, renal and hepatic failure), history of exposure to ionising radiation or neoplasia in the cervical region, history of malignancy and thyroid surgery, hypothyroidism caused by postpartum thyroiditis, pregnancy and lactation, neurological and psychiatric disorders.

### Power analysis and sample size

**Fig. 1:**
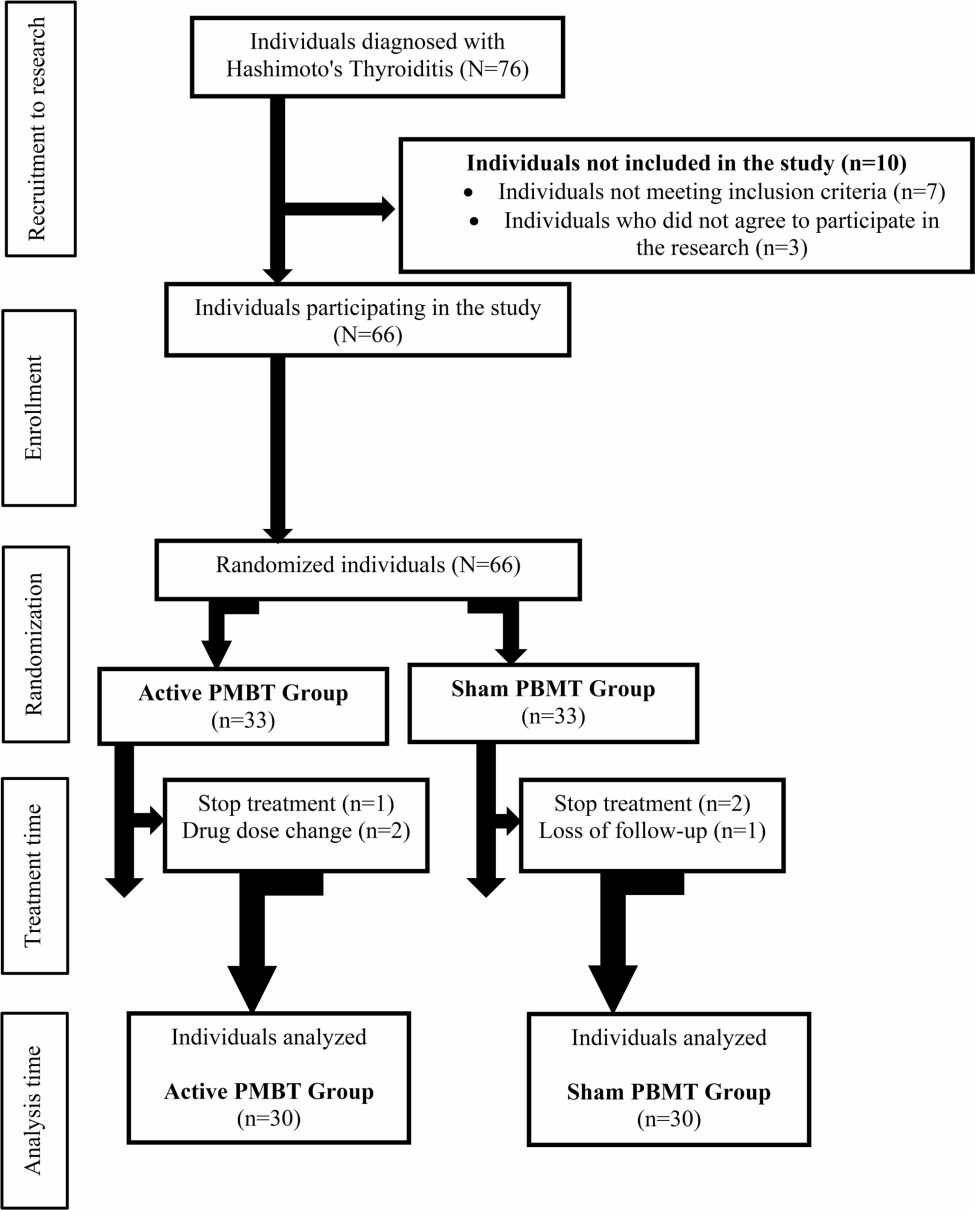
Working flow diagram

A priori power analysis was performed using G*Power software version 3.1.9.7 (G*Power, Universität Düsseldorf, Düsseldorf, Germany). The primary outcome parameter was accepted as fatigue, and the effect size was determined according to Cohen’s (d = 0.8). Using a two-tailed hypothesis, it was found that a patient group of at least 52 individuals was required to obtain 80% power in a sample size calculation with 95% reliability. To account for possible dropouts, seventy-six people were initially assessed. During the study, sixteen participants withdrew for various reasons, resulting in a final sample size of *n* = 30 in each group (Fig. 1).

## Methods

### Randomization

The study was conducted as a randomised, single-blind, sham-controlled. At the beginning of the study, 76 patients with HT who were being followed up in outpatient clinic were evaluated. These patients were invited to the face-to-face study during outpatient clinic visits. Sixty-six patients who accepted the study and met the inclusion criteria were determined to be eligible for the study and randomization was performed. The flow diagram of the research is shown in Fig. 1. Randomization was performed by generating a random number using the Randbetween function of Microsoft Excel. Participants were assigned to two groups: active PMBT and sham PMBT. Thirty patients in each group were included in the study analysis. The patients in the groups did not have information with regard to which group they were included in. Clinical evaluations were performed before treatment and at the third post-treatment month.

### Photobiomodulation therapy

All patients diagnosed with HT in the study were receiving LT4 treatment. In the PMBT group, in addition to thyroid hormone replacement therapy, a continuous wave GaAIAs type diode laser device (Intelect^®^ Mobile Laser, Model No: 2779, Production Year: 2016; Chattanooga Group) was also applied to the thyroid gland in a 0.07 cm² treatment area. Continuous mode at 850 nm wavelength, 100 mW output power, 1.43 W/cm^2^ power density and 28.57 J/cm^2^ energy density (28.57 J/cm^2^ =100 Mw x 20 s/0.07) were used during the PBMT.

Before each treatment session, the borders of the thyroid gland were marked with ultrasound by an endocrinology specialist. Eight target points (superior, mediolateral, inferior borders of the right and left thyroid lobes, right and left side of the isthmus) were demarked with a surgical pen. PMBT was applied to the marked areas on the thyroid gland, which were approximately 1 cm apart from each other. During the treatment, the patient’s neck was placed in the extension position. At each marked point, 2 J radiant energy was applied for 20 s, keeping the tip of the probe perpendicular and in full contact with the skin. The cumulative dose was calculated as 96 J. The duration of each treatment session was approximately 3 min (160 s of total irradiation time), excluding preparation time. The treatment protocol was continued for three weeks (six sessions), two days a week (Table 1).

**Table 1:** Technical Parameters of Laser Equipment

Manufacturer	Chattanooga Grup (A Division of Encore Medical)
Model	Intelect^®^ Mobil Laser Model No:2779
Production year	2016
Number and type of emitters	GaAIAs type diode laser
Wavelength and bandwidth, nm	850 nm
Pulse mode, CW or Hz, duty cycle	CW (Continuous waveform)
Beam spot size at target, cm^2^	0.012
Treatment area, cm^2^	0.07
Power density, W/cm^2^	1.43
Duration, sec	20
Energy density, J/cm^2^	28.57*
Radian energy, J	2 J
Number of irradiated points	8 target points
Irradiated area, cm^2^	0.56
Application technique	Full contact with skin
Number and frequency of treatment sessions	2 days a week for 3 weeks (6 sessions)
Total radiant energy during the whole treatment process, J	96 J

*Energy density=Output power x Application time/Application area

In the sham PMBT group, the probe was placed similarly to the treatment group. The screen of the laser device was active; however, the energy was set to 0 J and the power to 0 Mw, and the same procedures were also performed. During the present treatment, the patients were blinded with regard to which treatment they received.

### Evaluations

All clinical evaluations performed on the participants included in the study before and after treatment are listed as below. The primary outcome measure of the study was perceived fatigue level. Secondary outcome measures were pain severity, daytime sleepiness, sleep quality and behavioural status.

### Socio-demographic data form

Personal and disease information of all patients were recorded in the Patient Assessment Form. The form included the following information; age (years), gender, height (cm), weight (kg), body mass index (BMI) (kg/m²), occupation, marital status, educational status, medical history, family history, smoking, medication use, daily fluid consumption, exercise habits, diet status and complaints about the disease.

### Assessment pain severity and of the feeling of general fatigue

Pain intensity and general fatigue feeling of the individuals were evaluated by Visual Analogue Scale (VAS). In the assessment, a body diagram was used and the patient was asked to mark the area where they experienced pain. The level of pain felt by the participants at rest, during activity and at night was questioned. Individuals were asked to mark their fatigue level on the scale [16]. ‘0’ means ‘not tired at all’ and ‘10’means ‘very tired’.

### Assessment of fatigue level

Fatigue level in all patients was assessed by Fatigue Impact Scale (FIS) and Fatigue Severity Scale (FSS). The FIS is a forty-item scale indicating the consequences of fatigue on daily life such that it includes cognitive, physical and psychosocial subscales. The sum of the subscales ranges from 0 to 40 (physical and cognitive) and 0–80 (psychosocial); a higher score indicates a more severe impact of fatigue in daily life [17]. FSS assesses the degree of physical fatigue over the past week and includes nine items. Patients indicate how much they agree or disagree with the statements for each item on a seven-point Likert scale. FSS scores ≥ 4.0 indicate clinically relevant fatigue [18].

### Assessment of sleep quality and sleepiness

The Pittsburgh Sleep Quality Index (PSQI) was used to assess sleep quality based on participants’ sleep experiences in the past week. The PSQI is a self-administered questionnaire that has been proven to be a valid and reliable tool for measuring sleep quality and quantity. The PSQI consists of a total of seven components and nineteen questions, including subjective sleep quality, sleep latency, sleep duration, habitual sleep efficiency, sleep disturbances, use of sleeping medication and daytime dysfunction. The sum of the seven dimensional components gives global scores ranging from 0 to 21, with higher scores indicating poorer sleep quality. Those having score above 5 are considered as to have “poor sleep quality” and those having score 5 and below are considered as to have “good sleep quality” [19, 20].

Epworth Sleepiness Scale (ESS) was used to assess sleepiness. The ESS is a short, easy-to-administer questionnaire that subjectively assesses daytime sleepiness. The probability of falling asleep in the eight-day condition is determined by a scoring from 0 to 3 (0 = no chance of napping, 1 = slight chance of napping, 2 = moderate chance of napping, 3 = high chance of napping). The ESS score is the sum of eight item scores and ranges from 0 to 24. Higher ESS scores indicate greater daytime sleepiness [21].

### Assessment of behavioral status

Behavioural status of the patients was evaluated with Beck Anxiety Inventory (BAI) and Beck Depression Inventory (BDI), respectively. The BAI was designed by Beck et al., [22] to determine the frequency of anxiety symptoms in adults and adolescents. Turkish validity and reliability study was conducted by Ulusoy et al. The inventory consists of twenty-one items describing subjective, somatic or panic-related anxiety symptoms, respectively. Self-reports are based on a four-point Likert scale ranging from “not at all” to “severe” regarding the experience of this symptom in the past month. Higher scores indicate more severe levels of anxiety [23].

The BDI is a twenty-one-item self-assessment scale that measures symptoms of depression. The Turkish validity and reliability study of the inventory developed by Beck et al., [24] was conducted by Hisli [25]. Each item is scored from 0 to 3; the total score ranges from 0 to 63. It was stated that those having scores as seventeen and above were in the group at risk of depression. Depression levels: 0–9, minimal; 10–16, mild; 17–29, moderate; 30–63, severe [25].

### Statistical analysis

Statistical Package for Social Sciences (SPSS) Version 26 (SPSS Inc, Chicago, IL, USA) was used for data analysis. The socio-demographic characteristics of the individuals were analysed using descriptive statistics. The variables obtained from the measurements were expressed as percentage (%), arithmetic mean±standard deviation (Mean ± SD). The compatibility of the variables for normal distribution was evaluated by Kolmogorov-Smirnov test. Student’s-t paired test was used to analyse normally distributed dependent variables and Student’s-t independent test was used to analyses the independent data. Dependent variables that did not show normal distribution were evaluated by Wilcoxon signed rank test and independent data were evaluated by Mann-Whitney U test. Significance was accepted at *p* < 0.05 level.

## Results

This study was conducted to investigate the effects of PMBT applied in combination with LT4 treatment on fatigue, sleep quality and behavioural status in patients diagnosed with HT. In this study, sixty patients aged between 24 and 63 years were taken under consideration. Patients were randomly divided into two groups as active PMBT (*n* = 30) and sham PMBT (*n* = 30). Female/male distribution between the groups was homogenous.

Intra and inter inter-group comparisons of the physical and clinical characteristics of the patients in both groups were shown in Tables 2 and 3, respectively. No significant difference was found between the age and height of the groups. In intragroup analyses, a significant decrease was found in weight, BMI, waist, hip and neck circumference measurements in the active group at the end of treatment (*p* < 0.05). In the sham group, there was a significant increase in weight and BMI values and a significant decrease in neck circumference measurements (*p* < 0.05). In intergroup comparison, weight, BMI, waist and hip circumference evaluations showed significant improvement in favour of the active group (*p* < 0.05) (Table 3).

**Table 2:** Physical and clinical features of the groups

Physical Features	Active (*n* = 30)		t	*p*	Sham (*n* = 30)		t	*p*
	B.T Mean ± SD	A.T Mean ± SD			B.T Mean ± SD	A.T Mean ± SD		
Age (years)	42.90 ± 8.11	**-**			42.03 ± 7.82	**-**		
Length (m)	1.64 ± 0.08	**-**			1.61 ± 0.06	**-**		
Weight (kg)	71.75 ± 11.79	70.27 ± 11.51	6.556	< 0.001*	68.17 ± 13.01	68.40 ± 13.05	-2.516	0.018*
BMI (kg/m²)	26.60 ± 4.21	26.04 ± 4.03	6.325	< 0.001*	26.15 ± 4.66	26.24 ± 4.69	-2.519	0.018*
Waist (cm)	87.76 ± 10.12	86.68 ± 9.75	3.412	0.002^*^	89.22 ± 10.67	89.30 ± 10.68	-1.720	0.096
Hip (cm)	106.97 ± 8.28	106.37 ± 8.07	4.207	< 0.001*	105.35 ± 11.35	105.47 ± 11.37	-1.564	0.129
Waist/Hip (cm)	0.82 ± 0.07	0.81 ± 0.06	1.804	0.082	0.85 ± 0.10	0.85 ± 0.10	0.065	0.949
Neck (cm)	33.02 ± 2.40	32.53 ± 2.30	4.252	< 0.001*	34.33 ± 3.01	33.98 ± 2.92	2.971	0.006^*^

*SD* Standard Deviation, *B.T* Before Treatment, *A.T* After Treatment, *BMI* Body Mass Index

Student’s-t paired test; ^*^p < 0.05

**Table 3:** Comparison of the physical and clinical features of the groups

Physical Features	Between Groups	
	t	*p*
Age (years)	0.421	0.675
Length (m)	1.608	0.113
Weight (kg)	7.022	< 0.001*
BMI (kg/m²)	6.808	< 0.001*
Waist (cm)	3.633	0.001^*^
Hip (cm)	4.452	< 0.001*
Waist/Hip (cm)	1.737	0.092
Neck (cm)	0.814	0.419

*BMI* Body Mass Index

Student’s-t independent test; ^*^p < 0.05

### Pain severity and feeling of general fatigue

During rest, activity and at night pain intensity and general fatigue sensation were evaluated with VAS. Head, neck, back, lumbar, pelvic, knee, foot, shoulder and hand pain were detected in the evaluation of the presence of regional pain.

In intragroup analyses, significant decreases in the level of rest and activity pain and general fatigue sensation were found in both groups at the end of treatment (*p* < 0.05) (Table 4).The intensity of pain feeling at night did not change in the sham group (*p* > 0.05). According to the comparison between groups, pain intensity and general fatigue sensation after treatment showed significant improvement in favour of the active group (*p* < 0.05) (Table 5).

**Table 4:** Pain severity and general fatigue sensation values of groups

VAS	Active (*n* = 30)		z	*p*	Sham (*n* = 30)		z	*p*
	B.T Mean ± SD	A.T Mean ± SD			B.T Mean ± SD	A.T Mean ± SD		
Rest	3.97 ± 2.31	1.63 ± 1.77	-4.510	< 0.001*	3.90 ± 2.38	3.40 ± 2.21	-3.873	< 0.001*
Activity	5.13 ± 2.34	2.03 ± 1.97	-4.736	< 0.001*	4.17 ± 2.21	3.77 ± 2.06	-3.464	0.001*
Night	1.87 ± 2.82	0.67 ± 1.49	-2.825	0.005^*^	1.27 ± 2.27	1.20 ± 2.19	-1.414	0.157
Fatique	7.70 ± 1.80	2.93 ± 1.20	-4.820	< 0.001*	7.33 ± 2.06	5.97 ± 1.59	-4.604	< 0.001*

*SD* Standard Deviation, *B.T* Before Treatment, *A.T* After Treatment, *VAS* Visual Analogue Scale

Wilcoxon signed rank test; ^*^p < 0.05

**Table 5:** Comparison of pain severity and general fatigue sensation values of groups

VAS	Between Groups	
	z	*p*
Rest	-5.062	< 0.001*
Activity	-6.246	< 0.001*
Night	-2.752	0.006^*^
Fatique	-6.756	< 0.001*

*VAS* Visual Analogue Scale

Mann-Whitney U test; ^*^p < 0.05

### Fatigue level, sleep quality, daytime sleepiness and behavioural status

Significant improvement was found in fatigue level, sleepiness, sleep quality, anxiety and depression results after treatment in both groups (*p* < 0,05). In the intergroup comparisons, all clinical symptom conditions showed significant improvement in favour of the active group (*p* < 0.05) (Tables 6 and 7).

**Table 6:** Fatigue level, sleep quality, sleepiness and behavioural status assessment questionnaire results of the groups

	Active (*n* = 30)		t	*p*	Sham (*n* = 30)		t	*p*
	B.T Mean ± SD	A.T Mean ± SD			B.T Mean ± SD	A.T Mean ± SD		
FIS	69.83 ± 35.49	30.00 ± 22.28	7.544	< 0.001*	63.80 ± 22.12	58.80 ± 20.14	3.657	0.001^*^
FSS	42.50 ± 14.35	29.40 ± 13.82	5.098	< 0.001*	40.43 ± 12.17	37.57 ± 11.44	4.252	< 0.001*
PSQI	6.87 ± 2.99	4.70 ± 2.72	5.215	< 0.001*	6.40 ± 3.11	5.97 ± 2.55	2.282	0.030^*^
ESS	7.47 ± 4.38	5.07 ± 3.21	5.119	< 0.001*	8.30 ± 3.64	7.93 ± 3.58	2.083	0.046^*^
BAI	16.90 ± 12.85	10.37 ± 11.96	6.018	< 0.001*	14.53 ± 9.25	13.67 ± 8.98	3.112	0.004^*^
BDI	16.73 ± 10.12	9.37 ± 6.89	5.316	< 0.001*	15.07 ± 9.29	14.20 ± 8.18	3.372	0.002^*^

*SD* Standard Deviation, *B.T* Before Treatment, *A.T* After Treatment, *BAI* Beck Anxiety Inventory, *BDI* Beck Depression Inventory, *ESS* Epworth Sleepiness Scale, *FIS* Fatique Impact Scala, *FSS* Fatique Severity Scale, *PSQI* Pittsburgh Sleep Quality Index

Student’s-t paired test; ^*^p < 0.05

**Table 7:** Comparison of fatigue level, sleep quality, sleepiness and behavioural status assessment questionnaire results of the groups

Test	Between Groups	
	t	*p*
FIS	6.386	< 0.001*
FSS	3.852	0.001^*^
PSQI	3.795	< 0.001*
ESS	4.060	< 0.001*
BAI	5.056	< 0.001*
BDI	4.612	< 0.001*

*BAI* Beck Anxiety Inventory, *BDI* Beck Depression Inventory, *ESS* Epworth Sleepiness Scale, *FIS* Fatique Impact Scala, *FSS* Fatique Severity Scale, *PSQI* Pittsburgh Sleep Quality Index

Student’s-t independent test; ^*^p < 0.05

## Discussion

Thyroid dysfunction is characterised by signs and symptoms leading to loss of function in many organs and systems. Symptoms such as severe fatigue, muscle pain, proximal muscle weakness, cramps, joint tenderness and poor sleep quality are commonly observed in patients with HT due to the effects of the neuromuscular system. Although individuals receive adequate thyroid hormone replacement therapy, these symptoms persist permanently. In a study investigating the prevalence of neuromuscular symptoms, objective and subjective motor fatigue in euthyroid HT patients, it was reported that patients under thyroid replacement therapy were significantly increased pain and fatigue perception compared to healthy controls [5]. It has also been reported that fibromyalgia symptoms such as muscle pain and tenderness, fatigue, decreased exercise capacity and cold intolerance are similar to symptoms associated with endocrine dysfunction such as hypothyroidism [26]. Bazzichi et al. [26] found the presence of thyroid antibodies in a large percentage of fibromyalgia patients, resulting in a higher incidence of clinical symptoms in patients with thyroid autoimmunity. In our study, a significant percentage of HT patients receiving LT4 treatment was found to have symptoms such as persistent fatigue, weakness, daytime sleepiness, myalgia, muscle weakness and muscle cramps. Next, a large percentage of participants reported chronic pain in the neck, back and waist areas on the body diagram.

Studies examining the relationship between thyroid autoimmunity and chronic widespread pain have suggested that abnormal regulation of thyrotropin-releasing hormone may modulate abnormal pain perception in patients with autoimmune thyroiditis [27]. The role of inflammation and inflammatory cytokines in chronic pain processes is well recognized [28]. Many cytokines, including interleukin-1 beta (IL-1β), interleukin-6 (IL-6), interleukin-8 (IL-8) and tumor necrosis factor-α (TNF-α), and inflammatory by-products such as bradykinin, prostaglandins, growth factors, nitric oxide and substance P mediate chronic pain processes. The clinical implication is that control and treatment of inflammation will inhibit pain [29]. Inflammation has also been highlighted as a strong reason factor for fatigue in autoimmunity-related diseases. In addition, physiological conditions such as oxygen/nutrient supply, metabolism, mood, motivation and daytime sleepiness are also among the factors that influence the occurrence of fatigue. Therefore, it would make sense that strategies to reduce inflammation in people with autoimmune disease should be among the targets for treating fatigue [30].

In patients with HT, damage to thyroid tissue is accompanied by T cells, cytokines (TNF-α, IL-2, IL-6), chemokines and thyroid autoantibodies [31]. In studies, PMBT was found to inhibit gene expression. In additon, serum concentrations of proinflammatory cytokines such as TNF-α, IL-1β, IL-2, IL-6, IL-8 were also reduced by PMBT [32, 33]. Animal studies have shown the positive effects of PMBT in the form of reduced inflammatory reactions, reduced fatigue and improved muscle repair when optimal irradiation parameters are used [34, 35]. In the occurrence mechanism of these effects, it has been observed that PMBT reduces oxidative stress, improves mitochondrial function, stimulates mitochondrial respiratory chain, ATP synthesis and microcirculation [36, 37]. Based on the above data, we aimed that the anti-inflammatory properties of PMBT would be effective in reducing pain, fatigue severity and other related symptoms in patients with HT. Hofling et al., [14] reported that PMBT can improve thyroid parenchymal vascularisation and support thyroid function and autoimmunity in patients with hypothyroidism caused by chronic autoimmune thyroiditis and concluded that these effects are due to the anti-inflammatory and biostimulation properties of PMBT. However, the above the researchers did not provide any information about the symptoms of the patients. In this respect, our study contributed clinically to the relevant literature. As a result of our study, after PMBT applied to the thyroid gland combined with LT4 replacement therapy in HT patients, pain intensity and general feeling of fatigue decreased in active and sham groups. In the intergroup comparison, the active group showed more improvement than the sham group. This case may be attributed to the reduction of inflammation and improvement of mitochondrial function with PMBT administration. At the same time, the obtained results show that the effect also occurs as placebo.

Although a direct biochemical link in the mechanism between thyroid dysfunction and sleep function and its quality have not been identified, studies have reported that patients have poor sleep quality. Song et al., [38] showed that people with subclinical hypothyroidism has prolonged sleep latency, short sleep duration and lower sleep quality satisfaction compared to euthyroid individuals. In another study, it was reported that there is a strong relationship between hypothyroidism and insomnia, and many symptoms (such as muscle and joint pain, fatigue, cold intolerance, anxiety) that develop with thyroid dysfunction may trigger sleep problems [39]. Chen et al., [40] evaluated the efficacy of laser acupuncture in patients with chronic insomnia by considering the biostimulation effect of PMBT. The authors found a significant improvement in PSQI and ESS scores following PMBT intervention. As a result of the study, it was noted that PMBT may be an effective intervention to help relieve symptoms in patients who have difficulty falling asleep, wake up frequently at night, experience poor quality sleep and daytime sleepiness episodes [40]. In our study, after PMBT application to the thyroid gland, the active treatment group showed significant improvement in sleep quality and daytime sleepiness compared to sham treatment. We concluded that active PMBT improved sleep quality and daytime sleepiness with a greater effect on clinical symptoms (fatigue, pain) compared with sham treatment.

Thyroid dysfunctions and mood disorders, especially depression, are known to frequently coexist [41]. Many studies have pointed out that the reduced psychosocial well-being in patients with HT, despite euthyroid status [42]. In a case-control study conducted by Carta et al., [43], it was reported that the frequency of lifelong depressive episodes, anxiety problems and social phobia was higher in patients with HT compared to controls. It has also been remarked that the risk of depressive disorders in HT is independent of thyroid function as determined by routine testing [43]. Saravanan et al., [44] found significant deterioration in the psychological health of patients receiving LT4 replacement therapy compared with age- and sex-matched controls in a community-based study. In a meta-analysis of four studies of the effect of LT4 treatment in adult patients with subclinical hypothyroidism, no benefit was found for depressive symptoms [45]. Wekking et al., [46] stated that persistent impairments in neurocognitive function may be due to both autoimmunity and the awareness of having a chronic disease in itself. In addition, some authors have drawn attention to the relationship between these changes in behavioural status and mitochondrial dysfunction, which is characterised by decreased ATP biosynthesis. In recent years, it has been reported that transcranial laser application for the treatment of depression is effective on depression by correcting neurotransmitter abnormalities and stimulating mitochondrial function in the prefrontal cortex [47]. In another study, it was concluded that transcranial photobiomodulation using 945 nm light-emitting diode (LED) could clinically reduce anxiety and depression in university students with anxiety and depression [48]. In our study, laser application to the thyroid gland was performed using GaAIAs type diode laser at 850 nm wavelength. Anxiety and depression levels decreased in active and sham PMBT groups. In the intergroup difference analyses, the active group showed more improvement than the sham treatment group. Considering the relationships between the thyroid gland with the central nervous system and the function of mitochondria, we observed that PMBT administered to the thyroid gland can change the behavioural state with its anti-oxidation and anti-inflammatory stimulatory properties. In addition, the use of PMBT as a supportive treatment in patients with HT may have enhanced patients’ positive expectations and motivation, which may have contributed to the improvements observed in the sham group, reflecting a placebo effect.

### Strengths & Limitations

The randomized, sham-controlled design of this study allowed for differentiation between the specific effects of PBMT and non-specific (placebo) responses, thereby representing the study’s strong methodological aspects. In addition, the assessment of clinically relevant outcomes, including fatigue, sleep quality, and behavioral status, underscores the study’s clinical applicability and patient-centered relevance, representing key strengths of this work.

Despite the strengths findings, this study has some limitations. First, the effects of PMBT were evaluated over a relatively short period, limiting our understanding of its long-term efficacy. Second, improvements observed in the sham group may reflect placebo effects or patient expectations, emphasizing the need for further research to clarify treatment-specific effects. Taken together, these strengths and limitations emphasize both the clinical applicability of PBMT and the necessity of larger, longer-term studies to confirm and extend these findings.

## Conclusions

As a result of our study, it was determined that PMBT was effective in reducing clinical symptoms in patients with HT. In chronic autoimmune thyroiditis, PMBT has increased the motivation of patients as a supportive treatment option. Considering the ease of application and positive effects of PMBT, we can say that our study has new contributions to clinicians and may be useful for researchers.

## Data Availability

No datasets were generated or analysed during the current study.
